# Financialisation and the Reshaping of Private Healthcare: A Case Study in India

**DOI:** 10.1111/1467-9566.70041

**Published:** 2025-05-16

**Authors:** Benjamin M. Hunter, Indira Chakravarthi, Shweta Marathe, Susan F. Murray

**Affiliations:** ^1^ School of Social & Political Sciences University of Glasgow Glasgow UK; ^2^ Department of International Development King’s College London London UK; ^3^ SATHI Pune Pune India; ^4^ Independent Public Health Researcher

**Keywords:** corporate, finance, LMIC, market, privatisation, state

## Abstract

The incursion of the financial sector into contemporary politics, economies and societies has been noted, yet financialisation remains under‐explored in the sociological study of healthcare. Using the case of Maharashtra, India, and a qualitative research approach combining policy documents, witness seminars and in‐depth interviews, we offer an account of how financialisation has been facilitated and enacted in this sector. We analyse the restructuring of a healthcare system in favour of corporate chains and calculative logics, with concomitant changes including closure and takeover of smaller hospitals and re‐modelling of not‐for‐profit hospitals along corporate lines. We highlight ways in which such financialisation of healthcare, and its sidelining of health needs, has significance for care processes, professional dynamics, access to care and the parameters of regulatory systems.

## 1 Introduction

The contemporary context is defined by the ideological dominance of private finance and privatised economic accumulation, with an expanded role of finance in contemporary politics, economies and societies (van der Zwan 2014). Many commentators refer to a process of ‘financialisation’, described by economist Gerald Epstein as ‘the increasing role of financial motives, financial markets, financial actors and financial institutions in the operation of the domestic and international economies’ (Epstein 2005, 3). Although several alternative definitions exist, each varyingly oriented towards different scales, phenomena and disciplines (see Mader et al. (2020) for a summary of the definitional debates), Epstein's deliberate broad definition permits flexibility in its application to a range of sectors and contexts. Scholarly works on areas as diverse as the agrifood system, nature and the climate crisis, and conditional cash transfers have shown how financialisation has ‘transformed specific markets and how financial motives and values, specifically the prioritisation of returns to shareholders over other corporate and social values, have trickled down into everyday practices and processes’ (Storm 2018, 317). To date, this analytical lens is under‐utilised in the sociological study of healthcare.

Healthcare has become a major growth area for global finance, offering many new opportunities for investors: global acquisitions in healthcare stood at USD 60 billion in 2023 (Bain & Company 2024), exceeding total development assistance for health. The emerging scholarship on financialisation in this sector has so far focused either on broad sectoral changes, or on changes taking place within specific healthcare institutions, and in both cases is often located in high‐income settings (see below).

In this article, we examine how financialisation has ‘trickled down’ in India's private healthcare system and aim to advance the literature in three ways. First, we adopt a methodological approach to studying financialisation that combines policy analysis with an examination of practices and processes, blending these two complementary perspectives to provide a more detailed picture of changes that are unfolding as healthcare becomes subject to financial motives and values. In this, we respond to Eren Vural's (2017) call for research on ‘whether the financial imperatives advocated by PE (private equity) funds take root or not’ in healthcare systems (285). Second, we do this in the context of a middle‐income country in which healthcare is, to a large extent, financed and provided privately. It is thus typical of the large population middle‐income settings seen as offering the greatest opportunities for healthcare industries and private finance (Hattangadi and Kandhari 2023), and contributes to a decentring of healthcare sociologies away from Anglosphere and Western European contexts (Jeffery et al. 2023). Third, and perhaps most crucially, we show how processes of financialisation are reshaping healthcare systems, in this case by encouraging the growth of particular (corporate) forms of healthcare that distort or crowd out other forms of provision, and how these play out in the arenas of care processes, professional dynamics, access to services and regulatory systems.

## 2 Financialisation and Healthcare Systems

Academic literature on health and financialisation spans several overlapping strands. One is the relationship between financialisation in the economy, deteriorating social conditions and population health (Gouzoulis and Galanis 2021), and on the public funds available for social sectors such as health (Mendes and Marques 2009). Another points to the penetration of the finance industry into the fields of public health and global health (Hunter et al. 2025; Hunter and Murray 2019; Stein and Sridhar 2018) and the creation of specific instruments that facilitate this, such as impact bonds (Rowe and Stephenson 2016) and vaccine bonds (Hughes‐McLure and Mawdsley 2022). The third considers changes taking place within the pharmaceutical industry as the financial needs of shareholders take primacy and inform company processes for drug development, market targeting and valuation (Busfield 2020; Roy 2023).

Our study sits within a body of work that is largely distinct from the above three strands and that considers financialisation processes in the context of national healthcare systems. Prior to the advent of ‘financialisation’ analyses, a few scholars had analysed market‐oriented reforms of national health systems and subsequent involvement by financial institutions, focusing on high and upper‐middle income countries. Early examples centred on healthcare privatisation and commercialisation reforms of the 1990s and 2000s: for example, Iriart (2005) on the expansion of US and European insurance companies in Argentina; Chee (2008) on state support for, and entanglements with, transnational capital in Whiteside (2011) on Canada's public–private partnerships. The creep of financial institutions into healthcare was noted as an underlying factor in other transformative processes such as privatisation (Collyer and White 2011, 239).

More recently, scholars have drawn on theory about financialisation in order to understand a contemporary phase of healthcare reforms which eschew overt privatisations in favour of alternative public–private arrangements, and the opportunities and influence these create for financial institutions. This emerging body of work provides important and detailed insights into the sociopolitical context for financialisation processes, its form and scale within specific national settings and the actors involved. Many of these studies have centred on changes within European welfare states that have a stronger history of public provisioning; they include Bayliss' work on the partnering of private investors (via private healthcare providers and private finance initiatives) with public hospitals in the English National Health Service (Bayliss 2016), van Dijk et al. on the relationship between financially struggling hospitals and their banks and insurers in the Netherlands (van Dijk et al. 2023), and Cordilha on the use of financial markets to fund the social security system housing France's Assurance Maladie (Cordilha 2023). Research on financialisation outside European contexts, in settings where private provisioning is extensive, has been more limited. Key examples include studies documenting the finance‐oriented transformations in public healthcare in Brazil (Cordilha 2023), and the growth in ownership by domestic and international financial capital of healthcare providers (and insurers) in Turkey (Eren Vural 2017), Brazil (Cordilha and Lavinas 2018) and the United States of America (Bruch et al. 2024).

One of the questions left outstanding in the above research is how sectoral changes impact upon practices and processes within healthcare institutions, and how these grow and in turn transform the wider healthcare system. Prevailing methodological approaches based on analyses of policy documents, annual reports, financial reporting, and press media reports offer valuable but limited insights. A small number of researchers have used interviews to examine the processes and relations through which financialisation plays out in healthcare, for example, the interests and values that inform private equity investors operating in healthcare (Eren Vural 2017), and the careful framing of financial information and mobilisation of networks during negotiations between private hospitals and financial actors (van Dijk et al. 2023). Mulligan described the introduction of several techniques for cost‐cutting and profit‐extraction when a private health insurance company in Puerto Rico was taken over by a US investment firm, techniques that did ‘little to add value to the health system as a whole’ (Mulligan 2016, 50). Building on these contributions, and a wider body of sociological research on how people navigate privatisation processes (Collyer and Willis 2020; Hunter 2018), our research uses a mixed‐method qualitative approach. We generate and analyse data on the policy processes taking place at a macro‐level *and* data on the meso‐ and micro‐level practices and processes that take place within and between institutions.

## 3 Materials and Methods

Our case example takes place in private healthcare in India, building on an extensive literature on trends and social characteristics in this context (Baru 1998; Qadeer 2011). Private healthcare is a dominant form of provision in India, accounting for around 70% of outpatient care and 60% of inpatient care (Muraleedharan et al. 2020), and comprises a diverse array of informal and formal services. The dominance of this sector has manifested through the formation of public–private partnerships (Baru and Nundy 2008) and the expansion of foreign investment (Chakravarthi et al. 2017), corporate chains (Lefebvre 2010), medicities (Murray et al. 2016) and an influential medical–industrial complex (Baru 2018). This has been accompanied by the growing critical attention to what Jeffery refers to as ‘everyday corruption’ in Indian medicine, manifesting in networks of commercial relations and pressure amongst private providers to maintain high levels of service use and income (Jeffery 2024).

Our own mapping of the trajectory of government policy over the last 50 years and our case study aims to shift the analytical focus from commercialisation and privatisation to the opening of the door to global investors and to financialisation. We use findings from a detailed case study conducted across Mumbai and Pune, the two largest cities in Maharashtra state. This state is one of the most industrialised and urbanised in the country and is indicative of the path for India's modernisation. Mumbai, which has a population of 18.2 million, is the financial capital of India, whereas Pune, with a population of 4.6 million is an important manufacturing and Information Technology (IT) centre (population figures based on the most recent census data and growth rates—Government of India 2011). Both have growing middle‐class populations benefitting from service sector employment, improved living standards and rising life expectancy. Like India, more broadly, Maharashtra is notable for the dominance of its private healthcare provisioning: an estimated 2492 private hospitals in all as against 711 public hospitals (Kapoor et al. 2020).

### 3.1 Data Collection

We collated government and nongovernment (including consultancy) policy documents, and business press media reporting, via the Google search engine and professional networks, to establish the policy context for corporate healthcare in Maharashtra. This search was supplemented by manual searching of reference lists and citations. To obtain information on numbers and types of healthcare facilities, we consulted registers maintained by municipal corporations under the Bombay Nursing Homes Registration Act and the Maharashtra State Registrar of Companies.

To generate data on micro‐level practices within and between institutions, we conducted 45 qualitative in‐depth interviews with representatives from involved institutions in the two cities: senior health workers in private facilities, private hospital administrators, patient representatives, state government officials, private equity investors, an insurance administrator and an academic (see Table 1). These took place between October 2017 and December 2018 and were conducted in English, Hindi or Marathi, depending on the preference of the respondent. Respondents were chosen to reflect a broad range of experiences in the healthcare sector. Two interviews were conducted by telephone, the rest were carried out face‐to‐face. Most were audio‐recorded or detailed notes were taken during and following interview. Audio recordings were transcribed, translated into English prior to analysis, where required, and checked by one of the authors. Questions asked during interviews focused on the area in which the respondent was engaged, and typically covered changes taking place in healthcare services or funding in recent decades and their effects on the way in which services are provided and on medical practice and employment of medical professionals. This included changes in ownership and management and experiences with new areas such as health insurance.

**TABLE 1:** Background of interview respondents.

Characteristic	Number
Health workers representing medical specialities, general practice, nursing and practitioner‐owners of facilities	24
Private hospital administrators, primarily mid‐level managers in corporate hospitals and a CEO	10
Patient representatives	5
Senior government ex‐officials	2
Private equity investors	2
Insurance company administrator	1
Academic	1

We also conducted two witness seminars during 2018 on the topics of transformation in private healthcare since the 1980s and the regulation of private sector providers. These seminars are a specialised form of oral history group interview which has been used extensively by medical historians (Tansey, n.d.). Experts and first‐hand witnesses to specific events reminisce collectively with a view to documenting contemporary history events. Each witness seminar had 8–10 participants including leading clinicians, academics, activists and former state government officials. Each took place over a day, were audio‐recorded, and edited transcripts were published after the participants had an opportunity to check the content. The transcripts from our witness seminars are available in the public domain (Chakravarthi and Hunter 2019a, 2019b).

### 3.2 Data Analysis

We undertook a thematic analysis of transcripts from the witness seminars and interviews (or interview notes), with the assistance of NVivo software. An initial round of coding by three members of the research team assigned descriptive codes based on a reading of existing academic and policy literature and the interests of the team in macro‐ and micro‐level processes. All authors discussed coding processes during three‐weekly meetings, including the creation of additional codes and the grouping of codes in themes. A sample of transcripts coded by each member of the team were cross‐checked by other members of the team to ensure consistency. Initial findings from the research were ‘sense‐tested’ with project advisors who work in private healthcare and at preliminary dissemination consultations held in Mumbai.

This article reports the results following two further analyses. First, we used the policy and consultancy reports, business press media, and an initial reading of the witness seminar transcripts to produce a narrative overview of key policy and sectoral developments. This is reported in the first section of our Findings. Second, we undertook an additional round of thematic analysis, informed by academic literature on financialisation, where we sought to refine the initial coding framework. This generated two themes: on restructuring in ownership and practice, and on concomitant changes in how healthcare is provided. Much of the data contributing to these themes came from our witness seminars and from interviews with investors, private hospital administrators, practitioner‐owners and former government officials. We include illustrative quotations from these sources in the second and third sections of the Findings, as well as information from the documentary materials discussed above.

### 3.3 Ethical Considerations

Ethics clearance for the research was obtained from institutional ethics committees of the research partners: Anusandhan Trust, Mumbai and King's College London. Witness seminar participants and interview respondents were informed of the aims of the research, the purpose of data collection and how the information they provided would be managed. Interview respondents were invited to speak at a time and place of their choosing, told about processes for anonymisation of interview data and informed that they could withdraw responses from the study at any time up until the end of the data collection period. Participation in the witness seminars was conditional on individuals consenting to attribution in publicly available transcripts, with the opportunity to check and edit comments prior to publication, as is the standard practice for witness seminars. All witness seminar participants and most interview participants provided written consent; two interviews conducted via telephone followed an oral process for consent.

## 4 Findings

### 4.1 A Domestic Policy Trajectory Towards Corporate Growth and Financialisation

Despite several decades of aspiration in favour of universal provision of curative, preventive and promotive health services, the context for much of India has remained one of low government funding for healthcare and insufficient capacity in public clinics and hospitals (Chakravarthi and Hunter 2019a, 43). In the absence of investment in a comprehensive public system for healthcare provision, a private healthcare sector became well established, in which largely allopathic curative care was offered by religious and charitable organisations and by practitioner‐owned small low technology for‐profit clinics and hospitals.

By the mid‐1990s there was a steady growth in demand for high‐end technologies such as cardiac bypass surgery, transplants, intensive care units and advanced diagnostics (Chakravarthi and Hunter 2019a, 41). This created an opportunity for larger for‐profit facilities to step into lucrative areas of provision for the wealthy and a growing middle‐class. The proportion of private healthcare enterprises categorised as hospitals in India rose from 15% in 2000–2001 to 26% a decade later (Hooda 2015). The new hospitals were established by local and NRI (nonresident Indian) doctors in collaboration with business investors.

Until the turn of the century the predominant mode of financing had been domestic, and foreign direct investment (FDI) was still very limited. For example, in Delhi the manufacturing conglomerate Escorts Group invested in the Escorts Heart Institute and Research Centre (Chakravarthi and Hunter 2019a, 41) and the owners of pharmaceutical company Ranbaxy in Fortis Healthcare (Arnold 2010). Another pharmaceutical company, Wockhardt, initiated Wockhardt Hospitals in Maharashtra State (Seetharaman 2013). In the southern Indian states, the Apollo chain was established (Gupte 2013; Hodges 2016). Soon this industry organised at the national level, and the Indian Health Federation was set up in 1998 by owners of large hospitals together with the Confederation of Indian Industry (CII) to assert their presence and lobby on their behalf. In the decade that followed, the federal government gave private hospitals industry status, whereas transnational management and accountancy firms such as McKinsey and PwC worked for CII on sector analyses and business reports, highlighting investment potential and making the case for public–private partnerships (Confederation of Indian Industry and & McKinsey 2002; PricewaterhouseCoopers 2007).

By the beginning of the 21st century, views on the desired client‐base were also widening. Trade in healthcare services, particularly the establishment and provision of super‐speciality hospitals and allied medical tourism, was now promoted by the federal and state governments for its potential to generate foreign currency. Health policy, previously a preserve of Ministries and Departments of Health, was becoming integrated with economic development plans. The National Health Policy 2002 encouraged medical facilities to provide services to users from overseas. The Minister of Finance's budget speech for the financial year 2003 called for India to become a world class Global Health Destination (Speech of Shri Jaswant Singh and Minister of Finance 2003, 1709). In June 2005, the Ministry of Tourism started the ‘M’ visas for medical tourists (Hazarika 2010).

A growing acceptance of foreign investment opened the passage from commercialised to financialised healthcare. From 2000, 100% FDI was permitted for all health‐related services under the automatic route. Between April 2000 and June 2019, the hospital and diagnostic centres went on to attract FDI worth USD 6.3 billion (India Brand Equity Foundation 2019b, 24). The list of international investors includes an array of development financing institutions, sovereign wealth funds and private equity firms (Chakravarthi et al. 2017; Marathe and Shukla 2024; Taneja and Sarkar 2023).

Active government support for the healthcare industry was important to facilitate its growth and its ability to offer returns on investment. Land and equipment was provided at subsidised rates to corporate hospitals (Chakravarthi and Hunter 2019a, 39). Medical tourism trade fairs, exhibitions, and conferences were supported in the Global North and South in partnership with private corporate hospitals, foreign governments and medical tour operators (Iyer 2004). Over the next decade plans were pursued by state governments in partnership with large private hospitals to attract foreign medical tourists for hip and knee replacements, eye and dental procedures, reproductive and surrogacy services, cancer treatment and organ transplants. Incoming medical travel had existed in Mumbai and some smaller cities on a limited basis for several decades, but now the Maharashtra state government established the Medical Tourism Council of Maharashtra and work began in earnest to directly compete with similar services in other countries, and in other Indian urban centres such as Delhi (Chakravarthi and Hunter 2019a, 60).

Nationally, the perceived synergy of healthcare and economic development was personified in the selection of Mahesh Sharma, politician and owner of Noida‐based Kailash Hospitals to lead the Ministry of Tourism (IMTJ 2015). In 2015, Advantage Healthcare India was launched, an annual event held in Noida and co‐sponsored by the Federation of Indian Chambers of Commerce & Industry (FICCI) and trade body Service Export Promotion Council. The federal government's Ministry of Commerce and Industry and FICCI commissioned a report entitled Medical Value Travel In India: Enhancing Value in MVT (IMS Health 2016). By 2017 India's National Health Policy document reported that there was now a robust healthcare industry with double digit growth, generating revenues and employment (Government of India 2017). At that point, the hospital industry in India was estimated to be worth INR 4 trillion (USD 61.8 billion) and was expected to double in value to INR 8.6 trillion (USD 132.8 billion) by 2022 (India Brand Equity Foundation 2019a, 5).

### 4.2 Restructuring of Ownership and the Penetration of Financial Practices

Processes of ownership restructuring are required for financial firms and investors to extract wealth. In India, the corporate hospital and its related diagnostics chains have been a necessary vehicle for the assimilation of healthcare as a site for financialisation. Apollo, founded in 1983, is widely considered India's first corporate hospital chain (Hodges 2016). Others such as Narayana, Fortis and Max followed in the early 2000s. By 2010, corporate‐owned hospitals were reported to be 10% of the total number of hospitals across the metropolises of Delhi, Mumbai, Kolkata, Chennai, Bangalore and Hyderabad (Ernst & Young figures, cited in India Brand Equity Foundation (2010)) and provided a far larger proportion of the inpatient beds. In 2015, the federal government's National Medical Directory stated that there were 42 private corporate hospitals in Maharashtra in 2015, of which 23 were in Mumbai and 18 in Pune (Hooda 2015, 27). The growth of corporate hospitals and diagnostics chains in Mumbai and Pune has become evident in the local newspapers, on internet sites, and in the suburbs and conurbations. Mumbai hosts Wockhardt, Asian Heart Institute, Metropolis, Surya Childcare, Jupiter, Global Hospitals, Lifewave hospitals, Healthspring, Pikale Hospital, Thyrocare and Metropolis among others, whereas in Pune there is Sahyadri, Aditya Birla, Shashwat Hospitals Private Limited, Gupte Hospital, Atharva Netralaya, Jupiter, Metropolis, Vitalife and AG Diagnostics Limited. There is also representation of companies established outside this state that have a pan‐Indian presence such as Apollo, Narayana and Cloudnine, and transnationals such as Columbia Asia.

Local chief executives in the sector are open in declaring that corporate healthcare had become important as a form of provisioning that can be extremely profitable: ‘Why are big groups putting in crores of rupees into healthcare? Because healthcare is a profitable business. Whether it is private, semi‐private or charitable, it makes money, and everybody wants to join in’ (corporate hospital CEO, in Chakravarthi and Hunter 2019a, 70). Nationally, five of the large multi‐speciality hospital chains—Apollo, Fortis, Narayana, Max India Limited and Healthcare Global Enterprises Limited—had combined reported revenues of INR 129 billion (USD 2.0 billion) in the year March 2017, an increase of about 80% over the course of a five‐year period from 2012 (Jain 2017). Single‐speciality chains have also emerged in ophthalmology, orthopaedics, oncology, dialysis, women's health (gynaecology‐obstetrics‐assisted reproduction), cardiology and imaging. In some cases, these were initially established by specialist clinicians, in other cases they were established by companies with commercial interests in a related area. Contacare, for example, is a Maharashtra‐based chain of eye hospitals set up by a company manufacturing ophthalmic equipment and lenses.

Ownership restructuring initially occurred as the profitable hospital chains looked to attract investments and raise additional funds. A private equity investor we interviewed indicated that almost all corporate hospital chains had received private equity investment; another explained that hospitals looking for investment approached leading investors in this field and claimed to currently have proposals from several hospitals ‘on my desk’. In Maharashtra, investments were made in diagnostics and hospital companies by India‐focused private equity funds such as: Asian Healthcare Fund in Mumbai‐based primary healthcare chain Wellspring Healthcare; by Tata Healthcare Fund in Pune‐based Lokmanya Hospitals; and by Sabre Partners in Oyster and Pearl Hospitals, Pune and Thyrocare, Mumbai. The country's largest healthcare corporates went further and sought to raise domestic and foreign capital by listing shares on a public exchange. Apollo listed in 2002, followed by Fortis in 2007. Narayana listed its shares in 2016, with its market capitalisation reaching USD 1 billion, and in 2018 it was ranked in the Fortune India 500, the top 500 corporations in India.

The corporate hospital chains underwent further ownership restructuring as joint‐ventures, mergers, and acquisitions were instigated by financial companies. The trajectory of a Maharashtra‐based chain, Sahyadri Hospitals, is illustrative. Since 2007, private equity funds such as ICICI Ventures and IDFC Alternatives have invested in and exited from Sahyadri. In 2017–2018 the chain reported net sales of INR 3.0 billion (USD 46.4 million) and a profit of INR 150 million (USD 2.3 million) (TEAM VCC 2019), and in April 2019 Everstone, a South‐East Asia‐focused private equity investor bought a controlling stake (TEAM VCC 2019). Mumbai‐based Surya Mother and Child has also seen turnover in its investors: it received equity investment from US‐based OrbiMed in 2013, then Mumbai‐based SeaLink Capital bought OrbiMed's stake in 2018 as part of a larger package of investment in the hospital chain (Rai 2018). In some cases, the scale of transactions are considerable: pan‐India chain Fortis was acquired for INR 40 billion (USD 1.1 billion) by one of the largest healthcare groups in the world, IHH, itself listed on the Bursa Malaysia and the Singapore Stock Exchange and counting a range of public and private global investors among its major shareholders (Altstedter 2018).

The ramifications of this type of investment were a step change from the existing for‐profit commodified healthcare. Investment capital requires movement and the mechanisms to achieve this were described to us as follows by the investors we interviewed. Growth in company value is required and this is gained through the rapid expansion of healthcare chains. Hospital revenue is used in the meantime to service debts and extract management fees. Typically, there will be a 3–7‐year investment cycle during which the number of facilities in a chain can be increased, with potential for a doubling or tripling of the number of facilities. With this, equity can be sold to new investors at a substantially increased value.

The need to meet investor expectations and perpetuate the investment cycle results in a variety of strategies to increase company value. Much publicity is devoted to the private sector's potential to increase bed numbers and create new healthcare infrastructure. But according to private equity respondents, the capital‐intensive nature of healthcare provision limits opportunities to achieve quick scale‐up through greenfield developments. Chains actually find it simpler to expand by acquiring existing hospitals. Doing so bypasses the challenges that arise with establishment of a new facility such as recruiting health workers and attracting user flows. A hospital CEO explained to us that they had put together a list of 20–30 hospitals in Pune that were possible targets for acquisition, prioritising those in debt distress, ‘I went and visited five of them. Some may have taken a loan of Rs 15 crore [USD 1.8 million] and cannot service it […] “you take the hospital, service my loan”’. Company restructuring offers alternative methods to increase value. An interviewee noted that they achieved significant returns on an investment in a hospital chain not only because the chain had doubled in size during their time as an investor but also because they had instigated formation of a separate company to take ownership of the service provider's land. This re‐structuring of liabilities created a new tradeable asset while increasing value for investors.

### 4.3 The Reshaping of Private Healthcare Provision

In a sector marked by competition for staff and users, and in which hospitals are expected to function as autonomous rival units, the profitable organisation of healthcare is paramount. One manifestation has been the growth of hospital administration as a field of expertise with specialist Masters in Business Administration (MBA) qualifications, as seen in the USA and elsewhere. From the mid‐1990s in India, the large private hospitals began to appoint those trained in finance, commerce, hospital or human resource management as CEOs replacing those with medical backgrounds (Chakravarthi and Hunter 2019a, 46). Calculative logics—an instrumentalist emphasis on quantified (financial) considerations in decision‐making—became more overt and were normalised within the institutional infrastructure. Billings departments were established to organise the costing of every episode and action. Marketing departments looked after branding, networking and advertising requirements. As a specialist with extensive experience working in corporate settings observed: ‘what happens is that revenue needs to be generated from everything […] they suddenly had to have a corporate office in the hospital, so the corporate office comes, a marketing department comes, and the billing department grows from one or two persons to a large department. Everything would cost money and whatever does not generate revenue is stopped’ (Chakravarthi and Hunter 2019a, 19).

The financial interests of investors intensify the pressures placed on hospital administrators, shaping the ways that their success or failure are defined. Senior management staff may be appointed on a profit‐sharing basis or given other related incentives, with revenue targets at the level of the hospital filtering down into monthly targets for individual departments. A hospital administrator described the mindset this instils in managers:

Suppose this department has a particular target. Suppose you meet that target, say INR 1 crore revenue for this month. So, [a portion of] anything beyond INR 1 crore, will go to your management. Whatever it is 10%, 1%, 2%, whatever. […] So, they don't bother about anything [else], just increase the revenue’.

Interviewees noted that specialist services and wards considered unprofitable can be closed, and procurement of new technology is based on estimated ability to recoup costs and generate new income. The effects on employment relations and clinical practices within the corporate hospitals have been considerable. Better‐resourced facilities and advanced technologies in corporate hospitals are a significant draw and well‐regarded specialists are offered incentives to join corporate hospitals with accompanying expectations of future revenue generation, creating an environment that promotes greater medical intervention. Pressure for high ‘conversion rates’ (the proportion of a specialist's outpatients admitted for inpatient care), and an institutional focus on inpatient ‘hospitality’ and advanced technologies, has normalised prolonged hospitalisation and large batteries of tests. The growing use of private insurance facilitates this.

The nongovernment segment of the Indian healthcare system was already substantial but it is now undergoing restructuring due to the processes outlined above. Below, we identify two of those shifts: the closure, merger and sale of smaller for‐profit hospitals, and the re‐modelling of not‐for‐profit hospitals along corporate‐friendly lines.

In past decades, newly qualified health workers in India could join public facilities or, in the case of doctors, could join or establish their own small private facility with an out‐patients clinic and a few beds, commonly referred to as a ‘nursing home’ (Chakravarthi and Hunter 2019a, 13–17). This latter option was the career ambition for many, with expectations of slow growth, family succession and the rewards of long‐term relationships with local patients. Incomes were based on fee‐for‐service models and cash‐for‐referral networks (the latter are known as ‘cut practices’). But by the early 2010s these nursing homes and small hospitals were in trouble. Given the poor state of maintenance of registers, it is difficult to obtain the exact numbers but the Bombay Nursing Homes Association reported in 2012 that one‐fifth of the 650 nursing homes registered with them had shut down, most of these in south Mumbai, that others were winding up or on the way to closure, and no new nursing homes had been established in the preceding 5 years (Rao 2012). The magazine of the Association of Medical Consultants was carrying advertisements for the rent or sale of plots formerly occupied by nursing homes (Rao 2012), and trade magazines carried advice on how to survive (Healthcare Executive 2016).

Pressures were multiple, and according to many of our interviewees and seminar participants, the erosion of the small private hospital/nursing home sector stemmed from a combination of factors: the prohibitive cost of buying land, particularly in Mumbai; the expense of procuring and maintaining the more sophisticated technologies offered in the larger facilities; difficulties recruiting medical staff due to competition with corporate chains and the administrative burden of complying with government regulations. Respondents noted the relative ease with which large corporate hospitals could sidestep or overcome such difficulties.

Another factor was health insurance, a strand of financial services that is also intensely engaged in creating new Indian markets (Murray 2024). Health insurance arrangements often provide important stable income for large private sector providers while working against small hospitals: ‘The problem with insurance is that they reimburse same procedure at different rates in different hospitals. They ask for a procedure to be done here at a lesser rate than in say a corporate hospital’ (Clinician managing a small hospital in Pune). The minimum standards set for facilities and the administrative requirements of insurance claims were also difficult for small providers to meet. In contrast, corporate hospitals could establish billings departments for processing large numbers of claims, and had the financial and technical resources to offer desirable ‘cashless’ insurance services in which the insurance company settles the hospital bill directly.

The second shift involves a remodelling of the not‐for‐profit sector. The private healthcare sector in Mumbai, and to a lesser extent Pune, had historically been characterised by the prominence of not‐for‐profit charitable institutions that provide all levels of clinical care (Chakravarthi and Hunter 2019a, 18–20). In 2017, there were 76 of these ‘trust hospitals’ in Mumbai and 57 in Pune. They were typically established in the mid‐late 20th century by religious groups or industrialists. Annual expenditure must exceed INR 500,000 (USD 7700) and registration as ‘state‐aided public trusts’ is required under the 1950 Bombay Public Trusts Act. These trusts receive government subsidies and concessions on land, electricity, building rules, income tax and import of medical equipment, and in return are expected to provide free and subsidised medical care to poorer populations, whereas functioning in a generally philanthropic spirit characterised by low fees for all paying users.

Trust hospitals found themselves competing with the corporates for an increasingly aspirational middle‐class clientele but were constrained by obligations to behave charitably. Some hospitals have ‘sickened’ in this environment, and became financially unviable (Chakravarthi and Hunter 2019a, 19). Management practices imitating features of the corporate hospitals are then used to turn around such hospitals. This includes introduction of nonmedical management cadres and business management practices, prioritisation of revenue generation, and with that prioritisation of the services that are most lucrative. Study respondents pointed to shifting attitudes amongst trustees as the initial philanthropic vision of founders has been replaced by a greater business‐orientation amongst subsequent generations: they ‘have studied in foreign [countries], so they think in those lines, in business management and all, they think differently, they think it's a business’ (former government official interviewee). Religious principles of charitable giving have weakened in the face of financial pressures and there have been increases in cost of services provided. Trust hospitals have become increasingly reluctant to provide free or subsidised care to poor patients, and some reportedly use fictitious patients (gynaecologist interview in Mumbai), or care provided to the nonpoor families of employees, within their free care allocations (Chakravarthi and Hunter 2019a, 40).

Many trust hospitals have become targets for corporate takeovers (ibid., 24). Although trust‐run hospitals cannot be sold, many have entered into agreements with for‐profit hospitals or management companies to run their services. Vacant portions of land are leased out to for‐profit hospitals to construct new facilities in return for lease rentals and profit‐sharing arrangements with the trust, which itself continues its commitment to subsidised care for some poor patients. Well‐known Indian corporate hospital chains and management companies have made inroads in the Mumbai healthcare sector through this model, including Fortis (with Raheja Hospital), Ambani (Vasant Malti), Medanta (Parsi General), Apollo (Masina), Narayana (SRCC Children's Hospital) and Radiant Lifecare (Nanavati). Although a less prominent trend in Pune, examples in the city include Jehangir's agreement with Apollo and Sanjeevan Hospital's with Shashwat Hospital. These trends extend the business strategy of separating land from its use described earlier and mark an important departure from the founding ethos of these hospitals, further embedding corporate and investor cultures in the sector.

## 5 Discussion

Our findings show how corporate chains are a key vehicle for financialisation in middle‐income countries, where healthcare is largely financed and provided through private means. Corporate chains produce institutional structures of the size and legal standing required for entry, and wealth accumulation, by foreign investors. They are an emerging asset class that, as Hunter and Murray (2019) noted, build upon the ‘groundwork’ of commercialisation and privatisation in healthcare systems over the past 40 years (1266). Studying the formation of corporate healthcare in the context of Maharashtra complements accounts of financialisation that focus on corporate expansion in other middle‐income settings (Cordilha and Lavinas 2018; Hunter 2023; Eren Vural 2017).

Financial imperatives permeate the everyday of healthcare institutions and encourage financialisation in planning and practice. Our analysis points to new temporalities and valuative processes in the governance of healthcare institutions. The 3–7‐year entry and exit process for investment leads to repeated cycling of acquisitions, value‐addition and the sale of stakes. Indeed, since we concluded the research a series of investor exits, including Everstone's exit from Sahyadri, are evidence of this gathering pace (Bain & Company 2023). In Turkey's private healthcare sector, Eren Vural (2017) showed that pressure from investment cycles incentivised rapid growth as judged by ‘EBITDA’ (earnings before interest, tax, depreciation and amortisation). Our findings demonstrate some of the techniques used within hospitals to construct and augment this form of value, from the high‐level decisions around specialities and technologies that can be offered, down to the ‘banal everyday’ of financialisation (Bailey et al. 2019), such as billing processes. We have illuminated strategies of risk management—corporate chains and their investors mitigate financial risk by opting to acquire established facilities, re‐purpose land or partner with charitable hospitals, avoiding the up‐front costs and potential for unprofitability of greenfield sites (Hodges 2016, 154). Like Stein, we have shown how financialisation involves re‐making a sector such that risks to health equity are supplanted by the consideration of corporate/financial risk (Stein 2021).

Financialisation has not so much trickled down as swept through the private healthcare sector in Maharashtra, producing a wider cultural shift in which the narratives and practices of healthcare come to privilege investment value, managerial and marketing practices, calculations, and consumerism, affecting day‐to‐day work relations and practices. The creation of ‘financial distress’ appears to have been key: finance‐driven growth in one (corporate) part of the sector, induced distress in other (small and nonprofit) parts, and thereby created the basis for acquisitions and further corporate growth. In this our work complements recent findings by van Dijk et al. (2023) on the conditions for financialisation processes within two hospitals in the Netherlands. In the case of Maharashtra, it is financial distress playing out across swathes of the private healthcare sector that enables its subjugation to the interests and needs of investors.

The transformation of private healthcare into ‘saleable and tradeable assets’ (Hunter and Murray 2019, 1264) has significance for care processes, professional dynamics, access to care, and regulatory systems. There are direct effects upon what services are offered to, or pressed upon, users. Our Maharashtra study indicates that the processes through which profit is created for investors lead to downward pressure on resource use in pursuit of efficiencies, and upward pressure on fees demanded of users/payers. Clinical departments have been closed down and decisions around procurement are constrained by profitability criteria. Perhaps more concerningly, revenue targets have further incentivised unnecessary testing and treatment, with potential iatrogenic effects for users and their families (Brownlee et al. 2017). The associated cost inflation is likely to contribute to detrimental social and economic impacts in a country where catastrophic levels of health expenditure already affect 17% of households (Selvaraj et al. 2022).

Profitability criteria also affect staffing decisions. A recent review from the United States of America and other high‐income settings has linked private equity ownership to lower staffing levels, or lower‐skilled staff with some evidence of negative impacts on quality of care (Borsa et al. 2023). In Maharashtra, there are signs of a ‘re‐stratification’ within the medical profession to meet the needs of the corporate hospital market, with professional bifurcation between the much‐celebrated ‘star doctors’ who attract custom, and the lower ranks of precariously employed and heavily indebted medical practitioners (Marathe et al. 2020). This occurs in a context of declining public image for many doctors and growing violence towards them (Jeffery 2024), as users' frustration with a poorly regulated sector (see below) is directed towards frontline workers rather than the abstracted corporations they represent.

The incentives for private actors to create investable projects has led to the exclusion from curative healthcare for large sections of people. Growth of corporate chains has been focused in India's metro‐cities and more recently tier‐2 and tier‐3 cities, whereas smaller towns and rural areas have been neglected as populations are generally poorer and spread out. The urban poor and lower‐middle classes who lack private insurance also hold little attraction. Our findings indicate that even charitable hospitals are now looking to reduce services to the poor. The result has been a socially segmented system in which those with greatest health needs rely on a struggling and under‐resourced public system. Increasingly, in this scenario, the government response is to attempt to outsource to the private sector. Public funds have been channelled into basic health insurance schemes that try to tackle the exclusionary practices of private providers by covering some costs of private care (Hooda 2020). In pursuit of further growth, the industry is now calling for far more government funding to be directed into subsidies for corporate providers (NATHEALTH 2023) in order to expand profitable operations into rural areas.

Existing systems of public regulation in India appear ill‐equipped to cope with the twin pressures of profit‐pursuit and exclusion. Public regulation relies largely on professional statutory bodies and private hospital registers designed with practitioner‐owner practices in mind, not private equity‐owned corporate hospitals. The Clinical Establishments Act has (still) yet to be adopted by most states, and even where it has been adopted there is no requirement for disclosure of details on investors. Meanwhile the National Accreditation Board for Hospitals and Healthcare Providers (NABH), which sets out minimum standards for care, was a product of collaboration with the corporate healthcare industry and appears designed to segment and squeeze out smaller private providers from the market rather than address issues such as quality of services being provided (Hunter et al. 2022). Public and private insurers are expected to regulate pricing and ensure evidence‐based care, but have proved to be unable to do so, preferring to shift costs onto policyholders through the use of co‐payments, benefit ceilings, deductibles and the exclusion of high‐cost elements such as medications (Murray 2024). The indication is that ongoing financialisation, and the financial and market influence this affords corporate healthcare providers and private financial actors, is likely to inhibit attempts at public regulation, mirroring trends in the USA (Bruch et al. 2024).

In conclusion, our findings indicate how financial imperatives have been able to ‘take root’ (Eren Vural 2017, 285), while health needs are sidelined. As Roy (2023) noted of soaring valuations for pharmaceutical companies promising new, highly priced treatments, ‘life and health seemed to be not just uncoupled from conventional stock market metrics, but inversely related to them’ (133).

Financial actors are extending their influence in the healthcare systems of many countries. Financialisation in Maharashtra's private healthcare sector bears some resemblance to accounts from the USA, where public subsidy combined with aggressive private equity‐fuelled merger and acquisition strategies supported the growth of corporate providers (Appelbaum and Batt 2021). Yet there are important distinctions too. In the USA, the acquisition of not‐for‐profit providers, and their ‘conversion’ into for‐profit enterprises was a key component in early stages of financialisation (ibid.). As this was to some extent prevented by the legal terms of charitable hospitals in India, this has led to more imaginative ‘partnering’ approaches that ostensibly preserve that status while still facilitating cultural shifts of the kind we outlined above. In the United States of America, some corporate chains focused on acquiring community hospitals in rural areas in order to create captive markets without alerting anti‐trust regulators, whereas in Maharashtra, where anti‐trust concerns have less historical prominence, the first phase of the phenomenon has been one of very visible growth in urban areas. Marathe et al. (2020) noted how some interested parties see this as a process of ‘replacement of a low‐quality market segment by one with greater technology, life‐saving capacity and transparent systems’, but as we have shown, it also involves market consolidation and business models that are liable to concentrate supply amongst a small number of influential corporate chains and undermine health equity.

Financialisation unfolds in diverse and context‐specific ways. One of the key areas for future research will be the investigation of how financialisation processes impact upon the environment and delivery for healthcare in a wider range of settings including middle‐income countries, and how users and workers navigate this. Closer scrutiny of knowledges, networks and infrastructures that shape financialisation will be important for identifying opportunities to promote alternative models for healthcare financing and provision.

## Author Contributions

**Benjamin M. Hunter:** conceptualization (equal), data curation (equal), formal analysis (equal), methodology (equal), writing – original draft (lead), writing – review and editing (lead). **Indira Chakravarthi:** conceptualization (equal), data curation (equal), formal analysis (equal), funding acquisition (equal), methodology (equal), project administration (equal), supervision (supporting), writing – review and editing (supporting). **Shweta Marathe:** data curation (equal), formal analysis (equal), methodology (equal), writing – review and editing (supporting). **Susan F. Murray:** conceptualization (equal), funding acquisition (equal), methodology (equal), project administration (equal), supervision (lead), writing – original draft (equal), writing – review and editing (equal).

## Ethics Statement

Ethics clearance for the research was obtained from Institutional Ethics Committees of Anusandhan Trust, Mumbai and King's College London.

## Conflicts of Interest

The authors declare no conflicts of interest.

## Data Availability

According to the UK research councils' Common Principles on Data Policy, and Wellcome Trust's Policy on data, software and materials management and sharing, all data supporting this study are available via the UK Data Service: https://doi.org/10.5255/UKDA‐SN‐856569.

## References

[shil70041-bib-0001] Altstedter, A. 2018. “Malaysia’s IHH Healthcare Wins Fortis Hospitals Deal, Beats TPG‐Backed Manipal.” Hindustan Times, July 13. https://www.hindustantimes.com/business‐news/malaysia‐s‐ihh‐healthcare‐wins‐fortis‐hospitals‐deal‐beats‐tpg‐backed‐manipal/story‐WxdWx3Zhicf1EZmG4HD0BL.html.

[shil70041-bib-0002] Appelbaum, E. , and R. Batt . 2021. Financialization in Health Care: The Transformation of US Hospital Systems. Center for Economic and Policy Research.

[shil70041-bib-0003] Arnold, W. 2010. “Pursuing an Asian Health Network.” New York Times, August 26. https://www.nytimes.com/2010/08/27/business/global/27singh.html.

[shil70041-bib-0004] Bailey, S. , D. Pierides , A. Brisley , C. Weisshaar , and T. Blakeman . 2019. “Financialising Acute Kidney Injury: From the Practices of Care to the Numbers of Improvement.” Sociology of Health & Illness 41, no. 5: 882–899. 10.1111/1467-9566.12868. 30756403 PMC7027896

[shil70041-bib-0005] Bain & Company . 2023. India Private Equity Report 2023. Bain & Company.

[shil70041-bib-0006] Bain & Company . 2024. Global Healthcare Private Equity: Report 2024. Bain & Company.

[shil70041-bib-0007] Baru, R. V. 1998. Private Health Care in India: Social Characteristics and Trends. Sage Publications.

[shil70041-bib-0008] Baru, R. V. 2018. “Medical–Industrial Complex.” In Equity and Access, 75–89. Oxford University Press. 10.1093/oso/9780199482160.003.0004.

[shil70041-bib-0009] Baru, R. V. , and M. Nundy . 2008. “Blurring of Boundaries: Public‐Private Partnerships in Health Services in India.” Economic and Political Weekly 43, no. 4: 62–71.

[shil70041-bib-0010] Bayliss, K. 2016. The Financialisation of Health in England and Wales: Lessons From the Water Sector. Financialisation, Economy, Society, Sustainable Development (FESSUD).

[shil70041-bib-0011] Borsa, A. , G. Bejarano , M. Ellen , and J. D. Bruch . 2023. “Evaluating Trends in Private Equity Ownership and Impacts on Health Outcomes, Costs, and Quality: Systematic Review.” BMJ: e075244. 10.1136/bmj-2023-075244. 37468157 PMC10354830

[shil70041-bib-0012] Brownlee, S. , K. Chalkidou , J. Doust , et al. 2017. “Evidence for Overuse of Medical Services Around the World.” Lancet 390, no. 10090: 156–168. 10.1016/S0140-6736(16)32585-5. 28077234 PMC5708862

[shil70041-bib-0013] Bruch, J. D. , V. Roy , and C. M. Grogan . 2024. “The Financialization of Health in the United States.” New England Journal of Medicine 390, no. 2: 178–182. 10.1056/NEJMms2308188. 38197821

[shil70041-bib-0014] Busfield, J. 2020. “Documenting the Financialisation of the Pharmaceutical Industry.” Social Science & Medicine 258: 113096. 10.1016/j.socscimed.2020.113096. 32563788

[shil70041-bib-0015] Chakravarthi, I. , and B. M. Hunter . 2019a. Witness Seminar on Private Healthcare Sector in Pune and Mumbai Since the 1980s (Issue June).

[shil70041-bib-0016] Chakravarthi, I. & B. M. Hunter . 2019b. Witness Seminar on Regulation of Formal Private Healthcare Providers in Maharashtra.

[shil70041-bib-0017] Chakravarthi, I. , B. Roy , I. Mukhopadhyay , and S. Barria . 2017. “Investing in Health: Healthcare Industry in India.” Economic and Political Weekly 52, no. 45: 50–56.

[shil70041-bib-0018] Chee, H. L. 2008. “Ownership, Control, and Contention: Challenges for the Future of Healthcare in Malaysia.” Social Science & Medicine 66, no. 10: 2145–2156. 10.1016/j.socscimed.2008.01.036. 18329149

[shil70041-bib-0019] Collyer, F. , and K. White . 2011. “The Privatisation of Medicare and the National Health Service, and the Global Marketisation of Healthcare Systems.” Health Sociology Review 20, no. 3: 238–244. 10.1080/14461242.2011.11003086.

[shil70041-bib-0020] Collyer, F. , and K. Willis . 2020. Navigating Private and Public Healthcare: Experiences of Patients, Doctors and Policy‐Makers. Palgrave Macmillan.

[shil70041-bib-0021] Confederation of Indian Industry, & McKinsey . 2002. Healthcare in India: The Road Ahead. Confederation of Indian Industry & McKinsey.

[shil70041-bib-0022] Cordilha, A. C. 2023. Public Health Systems in the Age of Financialization: Lessons From the Center and the Periphery. Brill.

[shil70041-bib-0023] Cordilha, A. C. , and L. Lavinas . 2018. “Transformações dos sistemas de saúde na era da financeirização. Lições da França e Do Brasil.” Ciência & Saúde Coletiva 23, no. 7: 2147–2158. 10.1590/1413-81232018237.11422018. 30020371

[shil70041-bib-0024] Epstein, G. 2005. Financialization and the World Economy. Edward Elgar Publishing Ltd.

[shil70041-bib-0025] Eren Vural, I. 2017. “Financialisation in Health Care: An Analysis of Private Equity Fund Investments in Turkey.” Social Science & Medicine 187: 276–286. 10.1016/j.socscimed.2017.06.008. 28711284

[shil70041-bib-0026] Gouzoulis, G. , and G. Galanis . 2021. “The Impact of Financialisation on Public Health in Times of COVID‐19 and beyond.” Sociology of Health & Illness 43, no. 6: 1328–1334. 10.1111/1467-9566.13305. 34117649 PMC8441777

[shil70041-bib-0027] Government of India . 2011. List of Towns and Their Population | Maharashtra. https://censusindia.gov.in/towns/mah_towns.pdf.

[shil70041-bib-0028] Government of India . 2017. National Health Policy 2017 – India. JK Science.

[shil70041-bib-0029] Gupte, P. 2013. Healer: Dr Pratap Chandra Reddy and the Transformation of India. Penguin.

[shil70041-bib-0030] Hattangadi, A. , and J. Kandhari . 2023. Navigating Emerging Markets Healthcare Trends. Morgan Stanley Investment Management. https://www.morganstanley.com/im/en‐us/individual‐investor/insights/articles/navigating‐emerging‐markets‐healthcare‐trends.html#:~:text=Healthcare%20in%20emerging%20markets%20is,response%20to%20these%20changing%20dynamics.

[shil70041-bib-0031] Hazarika, I. 2010. “Medical Tourism: Its Potential Impact on the Health Workforce and Health Systems in India.” Health Policy and Planning 25, no. 3: 248–251. 10.1093/heapol/czp050. 19926658

[shil70041-bib-0032] Healthcare Executive . 2016. “Dear Nursing Homes: Here’s How to Stay Alive.” Healthcare Executive, March 8.

[shil70041-bib-0033] Hodges, S. 2016. “It All Changed After Apollo and Other Corporate Hospital Myths.” In Public Health and Private Wealth, 139–164. Oxford University Press. 10.1093/acprof:oso/9780199463374.003.0007.

[shil70041-bib-0034] Hooda, S. 2015. Private Sector in Healthcare Delivery Market in India: Structure, Growth and Implications (185). Institute for Studies in Industrial Development.

[shil70041-bib-0035] Hooda, S. 2020. “Health System in Transition in India: Journey From State Provisioning to Privatization.” World Review of Political Economy 11, no. 4. 10.13169/worlrevipoliecon.11.4.0506.

[shil70041-bib-0036] Hughes‐McLure, S. , and E. Mawdsley . 2022. “Innovative Finance for Development? Vaccine Bonds and the Hidden Costs of Financialization.” Economic Geography 98, no. 2: 145–169. 10.1080/00130095.2021.2020090.

[shil70041-bib-0037] Hunter, B. M. 2018. “Brokerage in Commercialised Healthcare Systems: A Conceptual Framework and Empirical Evidence From Uttar Pradesh.” Social Science & Medicine 202: 128–135. 10.1016/j.socscimed.2018.03.004. 29524868

[shil70041-bib-0038] Hunter, B. M. 2023. Investor States: Global Health at the End of Aid. Cambridge University Press.

[shil70041-bib-0039] Hunter, B. M. , D. McCoy , A. C. Cordilha , et al. 2025. Private Financial Actors and Financialisation in Global Health. 10.37941/RR/2025/1.

[shil70041-bib-0040] Hunter, B. M. , and S. F. Murray . 2019. “Deconstructing the Financialization of Healthcare.” Development and Change 50, no. 5: 1263–1287. 10.1111/dech.12517.

[shil70041-bib-0041] Hunter, B. M. , S. F. Murray , S. Marathe , and I. Chakravarthi . 2022. “Decentred Regulation: The Case of Private Healthcare in India.” World Development 155: 105889. 10.1016/j.worlddev.2022.105889. 36846632 PMC9941715

[shil70041-bib-0042] IMS Health . 2016. Medical Value Travel in India: Enhancing Value in MVT.

[shil70041-bib-0043] IMTJ . 2015. “India’s Four‐Point Strategy to Become a Leading Medical Tourism Destination.” LaingBuisson, November 30. https://www.laingbuissonnews.com/imtj/news‐imtj/indias‐four‐point‐strategy‐to‐become‐a‐leading‐medical‐tourism‐destination/.

[shil70041-bib-0044] India Brand Equity Foundation . 2010. Healthcare, November 2010. India Brand Equity Foundation.

[shil70041-bib-0045] India Brand Equity Foundation . 2019a. Healthcare, July 2019. India Brand Equity Foundation.

[shil70041-bib-0046] India Brand Equity Foundation . 2019b. Healthcare, October 2019, Vol. 24, no. October. India Brand Equity Foundation.

[shil70041-bib-0047] Iriart, C. 2005. “The Transnationalization of the Health Care System in Argentina.” In Commercialization of Health Care: Global and Local Dynamics and Policy Responses, edited by M. Mackintosh and M. Koivusalo , Palgrave Macmillan.

[shil70041-bib-0048] Iyer, M. 2004. “India Out to Heal the World.” Times of India, October 26. https://timesofindia.indiatimes.com/city/mumbai/india‐out‐to‐heal‐the‐world/articleshow/898987.cms.

[shil70041-bib-0049] Jain, M. 2017. “Private Sector’s Profits in Healthcare Soar as Indian Government Investment Stagnates.” Scroll, no. August: 1–8.

[shil70041-bib-0050] Jeffery, R. 2024. “Current Challenges for Doctors in India: Deprofessionalisation, Reprofessionalisation or Fragmentation?” Sociology of Health & Illness 46, no. 5: 795–814. 10.1111/1467-9566.13564. 36271825

[shil70041-bib-0051] Jeffery, R. , D. S. Jones , and K. Kumbhar . 2023. “The Historical Sociology of Medicine in India: Introduction to the Special Section.” Sociology of Health & Illness 46, no. 5: 791–794. 10.1111/1467-9566.13737. 38153853

[shil70041-bib-0052] Kapoor, G. , A. Sriram , J. Joshi , A. Nandi , and R. Laxminarayan . 2020. COVID‐19 in India: State‐Wise Estimates of Current Hospital Beds, Intensive Care Unit (ICU) Beds and Ventilators (Issue April). https://cddep.org/wp‐content/uploads/2020/04/State‐wise‐estimates‐of‐current‐beds‐and‐ventilators_24Apr2020.pdf.

[shil70041-bib-0053] Lefebvre, B. 2010. Hospital Chains in India: Coming of Age? Institut Francais des Relations Internationales.

[shil70041-bib-0054] Mader, P. , D. Mertens , and N. van der Zwan . 2020. “Financialization: An Introduction.” In The Routledge International Handbook of Financialization, edited by P. Mader , D. Mertens , and N. van der Zwan . Routledge.

[shil70041-bib-0055] Marathe, S. , B. M. Hunter , I. Chakravarthi , A. Shukla , and S. F. Murray . 2020. “The Impacts of Corporatisation of Healthcare on Medical Practice and Professionals in Maharashtra, India.” BMJ Global Health 5, no. 2: e002026. 10.1136/bmjgh-2019-002026. PMC7042603 32133190

[shil70041-bib-0056] Marathe, S. , and A. Shukla . 2024. “Perverse Development‐Examining German Development Finance Institutions’ Engagement in Private Healthcare Sector in India.” Development 67, no. 1–2: 100–107. 10.1057/s41301-024-00408-4.

[shil70041-bib-0057] Mendes, Á. and R. M. Marques . 2009. “O financiamento do SUS sob os ‘ventos’ da financeirização [The Financing of SUS in a Scenario of Financialization].” Ciência & Saúde Coletiva 14, no. 3: 841–850. 10.1590/S1413-81232009000300019. 19547783

[shil70041-bib-0058] Mulligan, J. 2016. “Insurance Accounts: The Cultural Logics of Health Care Financing.” Medical Anthropology Quarterly 30, no. 1: 37–61. 10.1111/maq.12157. 25331937

[shil70041-bib-0059] Muraleedharan, V. , G. Vaidyanathan , T. Sundararaman , U. Dash , A. Ranjan , and M. Rajesh . 2020. “Invest More in Public Healthcare Facilities: What Do NSSO 71st and 75th Rounds Say?” Economic and Political Weekly 55, no. 37: 53–60.

[shil70041-bib-0060] Murray, S. F. 2024. The Problem of Private Health Insurance: Insights From Middle‐Income Countries. Cambridge University Press.

[shil70041-bib-0061] Murray, S. F. , R. Bisht , and E. Pitchforth . 2016. “Emplacing India’s ‘Medicities’.” Health & Place 42: 69–78. 10.1016/j.healthplace.2016.08.004. 27693748

[shil70041-bib-0062] NATHEALTH . 2023. Rural PPP Hospitals: Private Operators Want Govt Help With Operational Costs, Permission to Fix Fee. https://nathealthindia.org/rural‐ppp‐hospitals‐private‐operators‐want‐govt‐help‐with‐operational‐costs‐permission‐to‐fix‐fee‐2/.

[shil70041-bib-0063] PricewaterhouseCoopers . 2007. Healthcare in India: Emerging Market Report 2007. PricewaterhouseCoopers.

[shil70041-bib-0064] Qadeer, I. 2011. Public Health in India: Critical Reflections. Daanish Books.

[shil70041-bib-0065] Rai, J. 2018. SeaLink Buys Out OrbiMed From Surya Children’s Medicare, Injects Fresh Funds. VCCircle. https://www.vccircle.com/sealink‐buys‐out‐orbimed‐from‐surya‐childrens‐medicare‐injects‐fresh‐funds.

[shil70041-bib-0066] Rao, M. 2012. Wounded by Strict Regulations. Hindustan Times, August 27.

[shil70041-bib-0067] Rowe, R. , and N. Stephenson . 2016. “Speculating on Health: Public Health Meets Finance in ‘Health Impact Bonds’.” Sociology of Health & Illness 38, no. 8: 1203–1216. 10.1111/1467-9566.12450. 27426224

[shil70041-bib-0068] Roy, V. 2023. Capitalizing a Cure: How Finance Controls the Price and Value of Medicines. University of California Press.

[shil70041-bib-0069] Seetharaman, G. 2013. “Khorakiwalas Are Now Successfully Rebuilding Their Hospital Network.” Business Today, January 20. https://www.businesstoday.in/magazine/features/story/khorakiwalas‐wockhardt‐hospitals‐39459‐2013‐01‐07.

[shil70041-bib-0070] Selvaraj, S. , K. A. Karan , S. Srivastava , N. Bhan , and I. Mukhopadhyay . 2022. India Health System Review. World Health Organization, Regional Office for South‐East Asia.

[shil70041-bib-0071] Speech of Shri Jaswant Singh, Minister of Finance . 2003. Introducing the Budget for the Year 2003–2004, 1704. Parliament of India Lok Sabha Digital Library, Finance Minister Budget Speeches.

[shil70041-bib-0072] Stein, F. 2021. “Risky Business: COVAX and the Financialization of Global Vaccine Equity.” Globalization and Health 17, no. 1: 112. 10.1186/s12992-021-00763-8. 34544439 PMC8451387

[shil70041-bib-0073] Stein, F. , and D. Sridhar . 2018. “The Financialisation of Global Health.” Wellcome Open Research 3: 17. 10.12688/wellcomeopenres.13885.1. 29552644 PMC5829462

[shil70041-bib-0074] Storm, S. 2018. “Financialization and Economic Development: A Debate on the Social Efficiency of Modern Finance.” Development and Change 49, no. 2: 302–329. 10.1111/dech.12385.

[shil70041-bib-0075] Taneja, A. , and A. Sarkar . 2023. First, Do No Harm: Examining the Impact of the IFC’s Support to Private Healthcare in India.

[shil70041-bib-0076] Tansey, T. n.d. What Is a Witness Seminar. Accessed April 27, 2020. http://www.histmodbiomed.org/article/what‐is‐a‐witness‐seminar.html.

[shil70041-bib-0077] TEAM VCC . 2019. “Everstone to Buy Controlling Stake in Sahyadri Hospitals.” VCCIRCLE, April 7.

[shil70041-bib-0078] van der Zwan, N. 2014. “Making Sense of Financialization.” Socio‐Economic Review 12, no. 1: 99–129. 10.1093/ser/mwt020.

[shil70041-bib-0079] van Dijk, T. S. M. Felder , R. T. J. M. Janssen , and W. K. van der Scheer . 2023. “For Better or Worse: Governing Healthcare Organisations in Times of Financial Distress.” Sociology of Health & Illness 46, no. 5: 926–947. 10.1111/1467-9566.13744. 38153907

[shil70041-bib-0080] Whiteside, H. 2011. “Unhealthy Policy: The Political Economy of Canadian Public‐Private Partnership Hospitals.” Health Sociology Review 20, no. 3: 258–268. 10.5172/hesr.2011.20.3.258.

